# Reliability and quality assessment of internet videos as guidance for dietary weight loss intervention: a cross-sectional study in China

**DOI:** 10.3389/fpubh.2026.1749849

**Published:** 2026-03-19

**Authors:** Shiqi Zhou, Yongjian Zhang, Xiaohong Chen, Qianqian Zhong, Fengqin Sun, Rui Xu, Zhongli Sun, Junying Sun, Lin Yang, Zhuoxian Xie, Qianle Zhang, Shiqi Yan, Yongjiang Cai, Hanyi Yu, Yanwu Xu

**Affiliations:** 1 School of Future Technology, South China University of Technology, Guangzhou, China; 2 Health Management Center, Peking University Shenzhen Hospital, Shenzhen, China; 3 Shenzhen Family Doctors Association, Shenzhen, China; 4 Shenzhen Nanshan District Medical Group Headquarters, Shenzhen, China; 5 Pazhou Lab, Guangzhou, China

**Keywords:** dietary intervention, internet videos, quality assessment, reliability assessment, weight loss

## Abstract

**Background:**

Obesity, a chronic condition affecting multiple physiological systems, poses major public health challenges. Dietary interventions are widely recognized as effective strategies for weight management. With over 4 billion Internet users worldwide, an increasing number of individuals rely on online platforms for health information. In China, TikTok, Bilibili, and Kwai are major channels for disseminating health-related content. However, the quality of dietary weight loss information on these platforms remains unclear.

**Objective:**

This study aims to assess the reliability and quality of the information in Chinese videos on dietary weight loss shared on the BiliBili, TikTok, and Kwai, three video–sharing platforms.

**Methods:**

We identified the top 100 dietary weight-loss videos on each platform in February 2024, resulting in a total of 300 videos. Video information quality and reliability were assessed using the Global Quality Score (GQS) and modified DISCERN (mDISCERN). Correlations between video quality and video characteristics were also analyzed.

**Results:**

The average GQS scores for BiliBili, TikTok, and Kwai were 2.04, 1.81, and 1.70, respectively, while the average mDISCERN scores were 2.01, 1.81, and 1.73. Median scores for both tools across all platforms were 2. BiliBili showed significantly higher GQS scores than TikTok and Kwai (*p* < 0.01 and *p* < 0.05, respectively). Regarding mDISCERN, BiliBili scored significantly higher than TikTok (*p* < 0.05), while the difference with Kwai was not statistically significant (*p* = 0.08). Nevertheless, none of the platforms achieved scores above 3, indicating generally low information quality and reliability. Significant positive correlations were found between video duration and both GQS (*r* = 0.41, *p* < 0.01) and mDISCERN (*r* = 0.32, *p* < 0.01). Additionally, strong correlations were observed between likes and saves (*r* = 0.90, *p* < 0.01), likes and comments (*r* = 0.92, *p* < 0.01), and saves and comments (*r* = 0.86, *p* < 0.01).

**Conclusion:**

While acknowledging limitations regarding cross-sectional design and specific sampling of single keyword and Chinese platforms, our findings highlight that the prevalence of low-quality videos on Chinese social media exposes viewers to significant risks of misinformation and inappropriate dieting. These findings underscore the need to promote digital health literacy and improve strategies for digital health communication.

## 1 Introduction

Obesity is a chronic disease, like hypertension and asthma, adversely effects affecting nearly on all physiological functions of the human body. The World Health Organization defines overweight and obesity as abnormal or excessive fat accumulation that poses a threat to health (1). About 2000 years ago, people began to pay attention to the impact of obesity on incidence and mortality rates (2). A substantial body of research has demonstrated that obesity increases the risk of developing diabetes (3), cardiovascular diseases (3, 4), or psychological disorders such as depression (5). Obesity is a serious public health threat. The core principle of any obesity treatment is to alter the balance between energy intake and energy expenditure, ensuring that energy expenditure exceeds energy intake. While medicinal interventions and bariatric surgery are common weight loss methods (6), they often require substantial resources and can be costly or inefficient for general population. However, a growing body of evidence suggests that dietary intervention is a critical factor in short-term and long-term weight loss (7–9), and can achieve ideal weight management without compromising health. Therefore, it is crucial for the public to assess the reliability of dietary intervention information gathered in daily life for weight loss.

Technological advancements have revolutionized the way people access medical information has undergone significant changes. Compared to traditional methods such as consulting doctors or reading medical books, more and more individuals prefer to seek medical information online (10, 11). As of 2021, the number of global Internet users has exceeded 4 billion (12), and studies have found that one-third of American adults use the Internet to learn about health issues (13). In recent years, video information has become increasingly popular. Statistics show that videos with the hashtag #weightloss on TikTok have been viewed more than 9.7 billion times worldwide (14), indicating that healthy weight loss is a global hot topic. Compared to traditional text, social media videos offer key advantages (15, 16). First, visual information is more easily absorbed and remembered than text, allowing for the rapid and cost-effective dissemination of health information to a broad audience. Second, the rich visual effects of videos can encourage users to spontaneously adopt healthy behaviors. However, due to the large number of content creators on video social platforms and less regulation of video content, the quality of online information on diet and weight loss varies significantly. Driven by the desire for engagement metrics such as likes and saves, creators often exaggerate effects to attract viewers. Consequently, this prioritization of popularity over accuracy means audience may inadvertently encounter incomplete or misleading information (17, 18). Therefore, quality assessment of medical science popularization videos online is essential to mitigate online misinformation and promote public health literacy.

In China, BiliBili, TikTok (Chinese version also named Douyin), and Kwai (Chinese version also named Kuai Shou) are three most popular video-sharing platforms. These platforms are currently the main online media for disseminating health information online, with a monthly activity level exceeding 100 million people (19). All information on these platforms is freely accessible and users only need to search for keywords of interest to view related videos. Previous studies have evaluated the quality of popular medical science videos on different topics on TikTok and BiliBili. Videos related to plastic surgery are considered reliable (20), while the quality of videos on liver cancer and gallstones is generally unsatisfactory (21, 22). Their findings indicate that the overall quality of medical popular science videos on these topics remains suboptimal. We also found that several studies have specifically analyzed the quality of weight loss videos on English-language platforms such as YouTube and TikTok (23, 24). However, to the best of our knowledge, there has been no research analyzing the quality and reliability of videos dietary weight loss intervention on Chinese video-sharing platforms. This gap is particularly concerning given the unique short-video ecology in China, where rapid algorithmic dissemination often outpaces content verification. In clinical practice, this specific digital environment has been increasingly linked to patients adopting unsafe weight-loss behaviors based on trending videos. In response to these challenges, this study aims to assess the quality of the most popular dietary weight loss videos on BiliBili, TikTok, and Kwai, providing an empirical basis for advancing digital health communications and enhancing public health literacy.

## 2 Method

### 2.1 Search strategy and data collection

**Figure 1:**
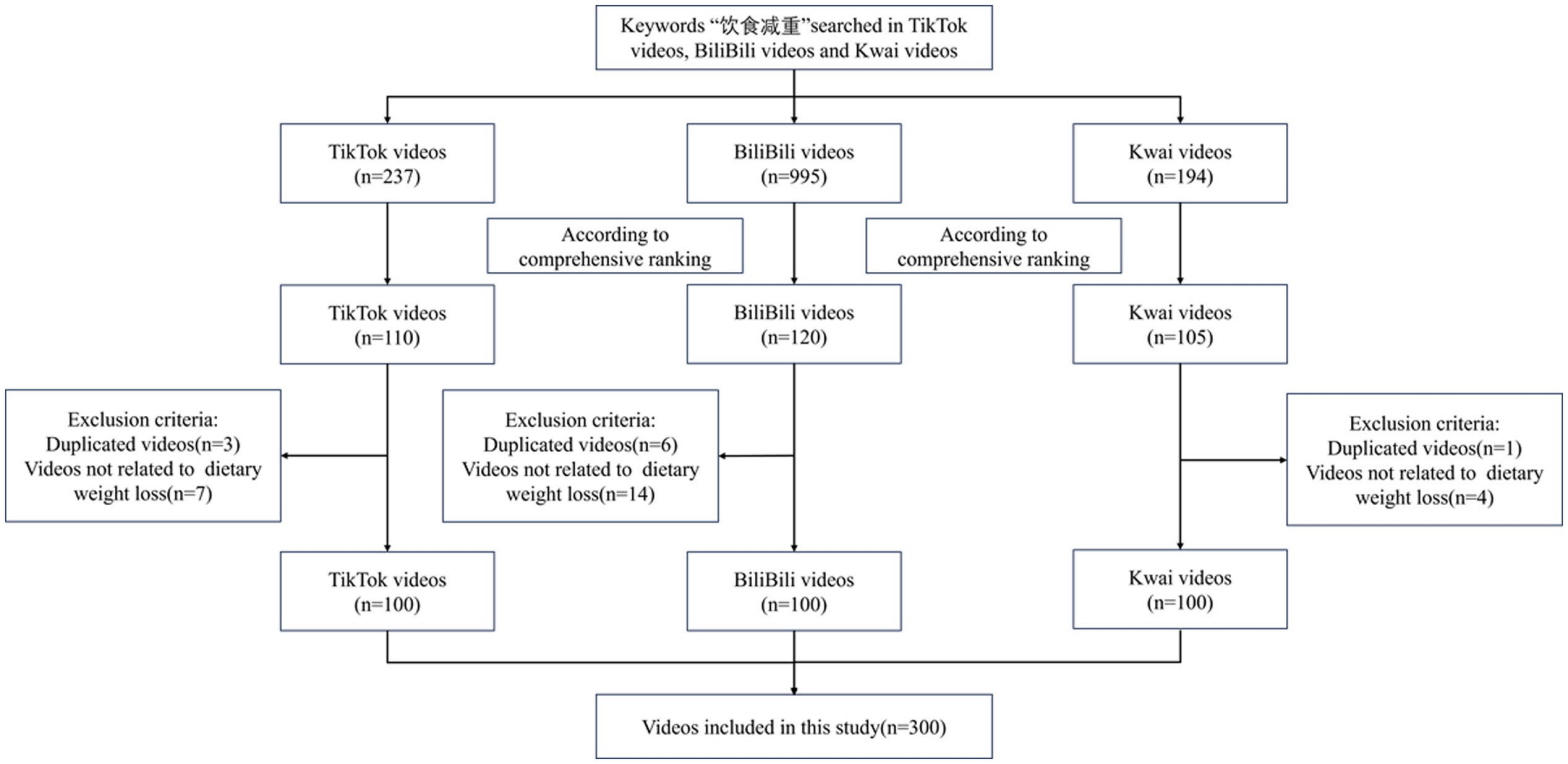
Flow diagram of video identification, screening, and inclusion for dietary weight-loss content across platforms.

As shown in Figure 1, in this cross-sectional study, we searched for videos related to “dietary weight loss” (饮食减重) on BiliBili (Chinese version 7.68.0), TikTok (Chinese version 28.6.0), and Kwai (Chinese version 12.0.40) on February 28th, 2024. For conceptual clarity, “dietary weight loss” was operationally defined as weight reduction strategies primarily achieved through modifications of dietary intake, including caloric restriction, macronutrient composition changes (e.g., ketogenic, low-carbohydrate, or low-fat diets), meal replacements, and time-restricted eating (e.g., intermittent fasting). Videos focusing primarily on exercise-only interventions, bariatric surgery, or prescription weight-loss medications were excluded.

To mitigate biases in video recommendations stemming from the personalized algorithms of the platforms, we conducted searches using newly created accounts on each platform without imposing any additional constraints. Videos were initially retrieved and ranked according to the platforms’ default recommendation algorithms. Subsequently, we screened the search results to exclude duplicate content and retained all videos related to the topic of dietary weight loss. Videos identified as part of a continuous serial topic were aggregated as a single one to ensure the completeness of the information provided. In accordance with established practices in recent digital health research, the top 100 eligible videos from each platform were selected to form the final dataset (21, 22).

To ensure the credibility of the video content, we verified the details of the video publishers. For instance, if a publisher was purported to be a medical professional from a hospital, we confirmed the accuracy of this information on the hospital’s official website. Beyond this, we also gathered video metadata for the videos, including the title, the publisher’s name, duration, and engagement metrics (the count of likes, saves, and comments). All collected data were securely stored in Excel (Microsoft Inc.) for further analysis.

To facilitate comparative analysis of information quality, we classified the videos according to two key dimensions. First, we categorized the videos into two groups based on their publishers: professional doctors and non-doctors. Second, the videos were classified into four content types: personal experiences, weight loss knowledge, advertisements, and other categories.

### 2.2 Ethical considerations

This study did not utilize any clinical data, human samples, or laboratory animals. All data were sourced from publicly available videos on BiliBili, TikTok, and Kwai, ensuring no personal privacy issues were involved. As the study did not engage directly with users, it did not require ethical review.

### 2.3 Video assessment

#### 2.3.1 Assessment criteria

We utilized the mDISCERN and the Global Quality Score (GQS) tools to assess the reliability and informational quality of the videos, respectively, the validity of which has been confirmed in previous studies (25–27). The mDISCERN is an adaptation of the DISCERN instrument proposed by Singh et al., and it evaluates the reliability of videos across five dimensions: clarity, reliability, fairness, references, and rigor (28). Each dimension is scored individually, with a total score ranging from 0 to 5; the higher the score, the better the reliability of the video. Compared to DISCERN, mDISCERN demonstrates improved convenience and accuracy in assessing video quality. The GQS, introduced by Bernard et al., was initially designed to evaluate the quality of health information on websites and has since been widely adopted for assessing the quality of video information (29). The GQS systematically evaluates the quality of videos based on informational quality, traffic, and usefulness, with a scoring range from 1 (very poor) to 5 (excellent).

#### 2.3.2 Video assessment procedure

We compiled the collected original videos and pertinent information, and then dispatched them to the professional raters involved in the scoring process. All participating video raters had extensive clinical expertise in dietary weight reduction. To minimize scoring discrepancies, the mDISCERN and GQS instruments were translated into Chinese to better align with the linguistic and contextual characteristics of Chinese short-video platforms. The detailed scoring criteria of both instruments, along with their corresponding Chinese translations, are presented in Supplementary Tables S1, S2, respectively. Subsequently, every rater thoroughly reviewed the Chinese-version criteria for GQS and mDISCERN. Prior to formal evaluation, a pilot scoring session was conducted on a subset of 10 videos to ensure a consistent understanding of the rating guidelines. For vlogs or humorous content containing personal narratives, raters were instructed to focus strictly on the extractable health assertions, disregarding entertainment elements. For content involving mixed languages or regional dialects, standard Chinese subtitles served as the primary reference for accuracy to ensure consistency among raters.

To prevent bias, a strict blinding protocol was enforced: platform watermarks were blurred, and raters were blinded to all engagement metrics and origin sources. Each video was then initially assessed by three primary raters (JS, QZ, and ZS). If their scores were unanimous, that score was final. For scores within a one-point range, the principle of majority determined the final score (e.g., mDISCERN scores of 1, 2, 2 and GQS scores of 3, 3, 2 would result in mDISCERN = 2, DQS = 3). If the score discrepancy exceeded one point (e.g., 2, 3, 4), two secondary raters (FS and RX) reassessed the video. If discrepancies remained, a tertiary grader (YZ) then held a final discussion to reach a consensus. Raters recorded their scores in a scoring spreadsheet; primary raters entered scores for all videos, while secondary and tertiary raters only recorded scores for videos they reviewed.

To further explore the difference in video ratings between professional physicians and the general audience, we engaged three raters (QL, SY, and ZX) with higher education but not medical expertise. They underwent the same standardized training and pilot scoring process to rate the videos based on the same criteria.

### 2.4 Statistical analysis

In this study, Data normality was first assessed using the Shapiro–Wilk test. As the data were not normally distributed, continuous variables are presented as medians with interquartile ranges (IQR), and categorical variables are presented as frequencies and percentages. Differences in continuous variables between two independent groups (e.g., doctor vs. non-doctor) were assessed using the Mann–Whitney U test.

To evaluate the reliability of the video assessments, we calculated the Fleiss’ Kappa coefficient to measure inter-rater agreement among the three primary raters. Additionally, to assess the divergence between professional and non-professional perspectives, Cohen’s Kappa coefficient was computed between the final scores of the two rater groups. A kappa value greater than 0.60 was considered indicative of substantial agreement.

Based on prior literature, we hypothesized *a priori* that longer video duration would be positively associated with quality scores, while engagement metrics (likes, saves, and comments) might show negative or non-significant associations. Accordingly, **S**pearman’s rank correlation analysis was utilized to examine the bivariate relationships between video quality scores (GQS, mDISCERN) and video characteristics.

To further identify independent determinants of video quality and reliability while controlling for confounding factors, multivariable linear regression analyses were performed separately for each platform. Prior to the regression analysis, categorical variables were recoded into binary dummy variables to ensure statistical validity. Uploader type was coded as a binary variable (1 = professional doctor, 0 = non-doctor). Similarly, given the limited sample size in certain subgroups, content category was reclassified into a binary variable: ‘Weight Loss Knowledge’ (coded as 1) versus ‘Other Content’ (including personal experiences, advertisements, and others, coded as 0).

All statistical analyses were performed using Python with the scipy and statsmodels libraries. Two-sided *p*-values < 0.05 were considered statistically significant. Given the exploratory nature of this study, we did not apply adjustments for multiple comparisons. Therefore, the multiple statistical tests performed may increase the risk of Type I errors (false positives) and marginal *p*-values should be interpreted with caution. For the regression results, coefficients (*β*), standard errors (SE), 95% confidence intervals (CIs), adjusted R^2^, and variance inflation factors (VIFs) were reported to evaluate model fit.

## 3 Results

### 3.1 Basic characteristics of videos

**Figure 2:**
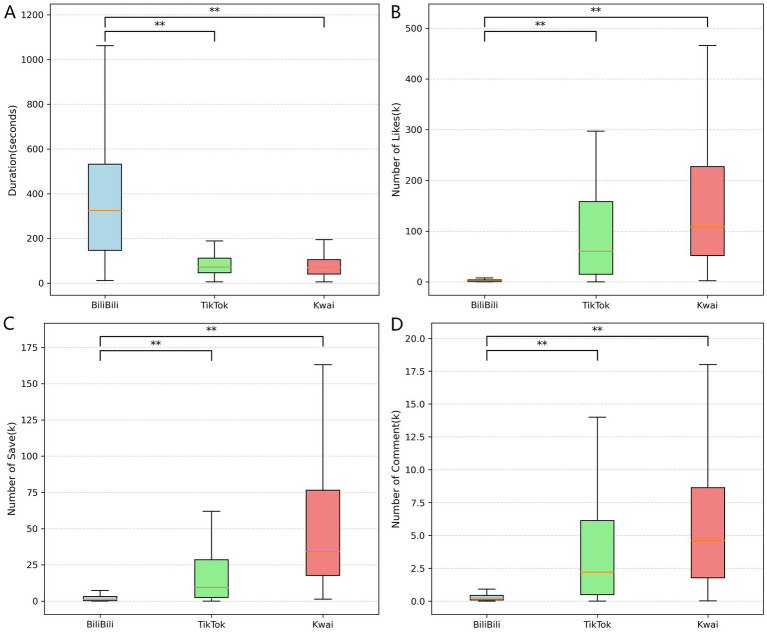
Boxplots of dietary weight loss videos characteristics across platforms. Panels show **(A)** Duration, **(B)** Likes, **(C)** Saves, and **(D)** Comments. In each boxplot, the center line indicates the median, boxes indicate the interquartile range (IQR, 25th–75th percentiles), and whiskers indicate 1.5 × IQR / (min–max). Pairwise comparisons between platforms were performed using Mann–Whitney *U* tests and *p*-values were unadjusted for multiple comparisons. *p < 0.05, **p < 0.01, ***p < 0.001.

We used the keyword “dietary weight loss” to retrieve videos on three platforms, respectively. After the screening, the top 100 videos in BiliBili, TikTok, and Kwai were retained. Table 1 summarizes the descriptive statistics of the video characteristics, and Figure 2 visually compares the data distributions across the three platforms using box plots. The video duration on BiliBili was significantly longer than that on TikTok and Kwai (*p* < 0.01). Specifically, the median duration for BiliBili was 325 s (IQR: 385 s), whereas TikTok and Kwai featured much shorter formats, with medians of 72 s (IQR: 65 s) and 70 s (IQR: 64 s), respectively. In contrast, TikTok and Kwai exhibited significantly higher engagement metrics (likes, saves, and comments) compared to BiliBili (*p* < 0.01), likely due to their shorter video formats. For likes, Kwai (Median: 108.51 k, IQR: 175.25 k) and TikTok (Median: 60.52 k, IQR: 143.25 k) far exceeded BiliBili (Median: 1.85 k, IQR: 3.35 k). A similar trend was observed for saves, where, where Kwai (Median: 34.03 k, IQR: 58.75 k) and TikTok (Median: 9.49 k, IQR: 26.03 k) were higher than BiliBili (Median: 1.15 k, IQR: 2.86 k). Regarding comments, Kwai videos had a median of 4.62 k (IQR: 6.85 k) and TikTok videos had a median of 2.20 k (IQR: 5.56 k), whereas BiliBili videos had a median of 0.23 k (IQR: 0.34 k).

**Table 1:** Characteristics of dietary weight loss videos collected from the three platforms.

Variable	BiliBili	TikTok	Kwai
GQS	2 (0–4)	2 (1–3)	2 (1–3)
mDISCERN	2 (0–5)	2 (0–3)	2 (0–3)
Duration (s)	325 (12–13,200)	72 (6–384)	70 (6–474)
Like (k)	1.85 (0.04–378.66)	60.52 (0.04–1765.23)	108.51 (2.42–1252.89)
Save (k)	1.15 (0.00–5.94)	9.50 (0.00–70.18)	34.21 (1.33–441.14)
Comment (k)	0.23 (0.01–4.42)	2.24 (0.02–207.85)	4.62 (0.02–84.23)

Data are presented as median (range). Group differences were evaluated using the Mann–Whitney U test, with *p* < 0.05 considered statistically significant.

**Figure 3:**
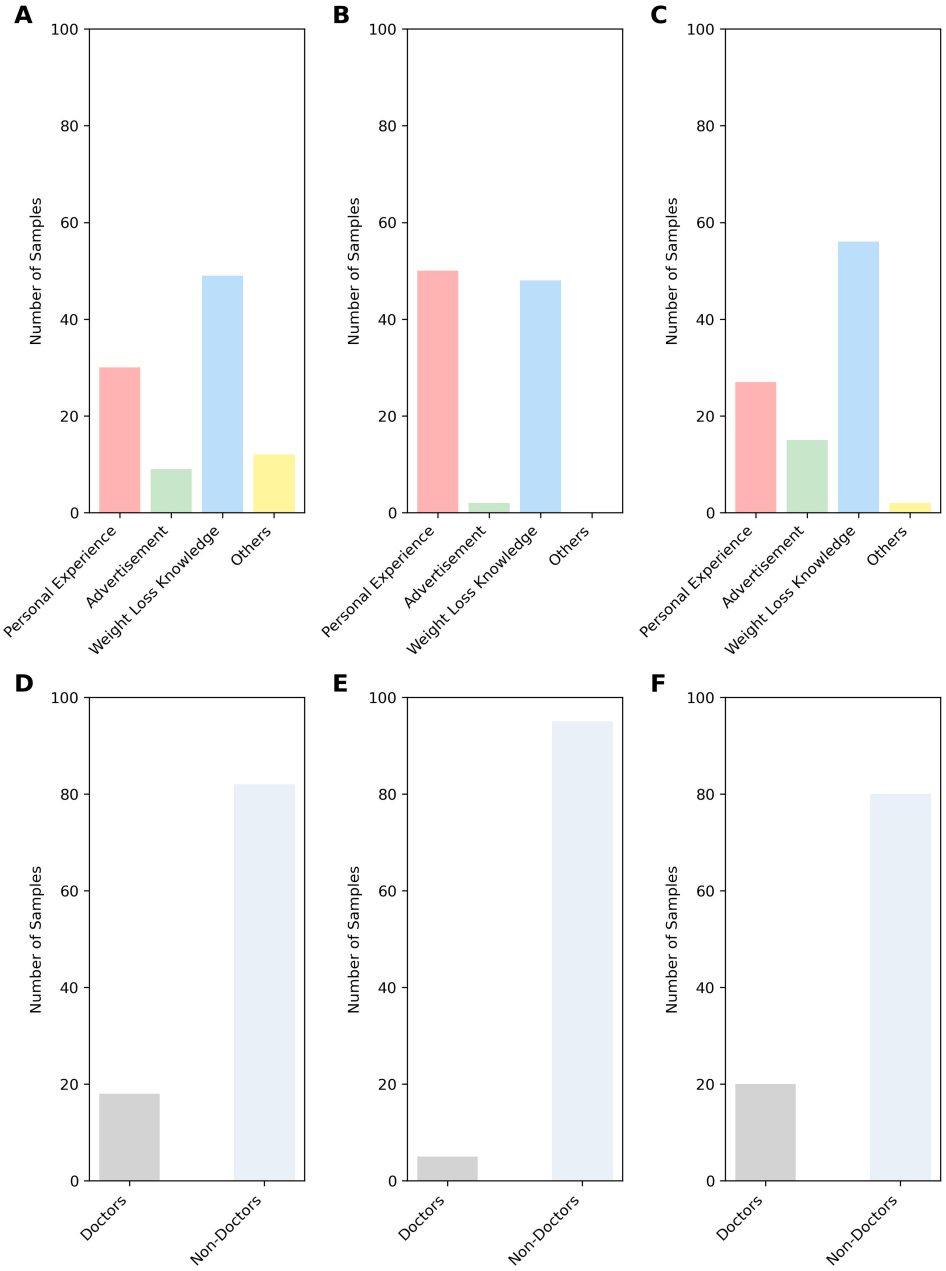
Distribution of dietary weight-loss videos by content category and uploader on different platforms. The top row shows video content categories on **(A)** BiliBili, **(B)** TikTok, and **(C)** Kwai. The bottom row shows uploader categories on **(D)** BiliBili, **(E)** TikTok, and **(F)** Kwai, respectively.

Figure 3 illustrates the distribution of videos by the uploader and content on the three platforms. Regarding sources, Kwai and BiliBili featured a higher number of videos from professional doctors (20 and 18, respectively) compared to TikTok (5). In terms of content, videos were classified into four categories: personal experiences, weight loss knowledge, advertisements, and others. “Weight loss knowledge” was the dominant category across all platforms (BiliBili: 49; TikTok: 48; Kwai: 56). Notably, TikTok had the highest volume of “personal experience” videos, reaching 50, which significantly exceeded the 30 on BiliBili and 27 on Kwai. Advertisements were also more frequent on Kwai and BiliBili, with 15 and 9 videos respectively, whereas TikTok contained only 2.

### 3.2 Video quality and reliability assessments

#### 3.2.1 Overall comparison across platforms

**Figure 4:**
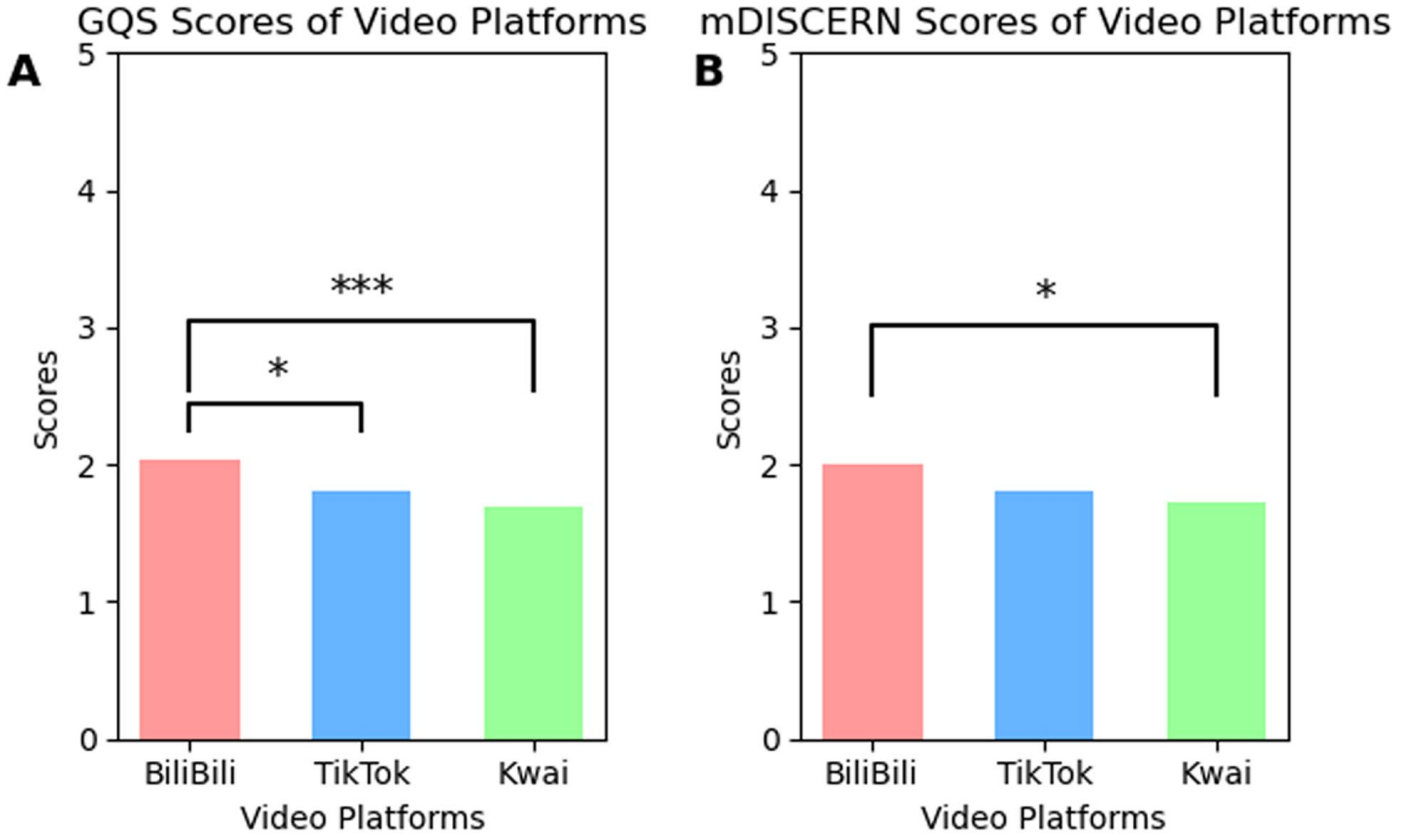
Comparison of **(A)** GQS and **(B)** mDISCERN scores determined by professional raters from three video platforms. Pairwise comparisons between platforms were performed using Mann–Whitney *U* tests and *p*-values were unadjusted for multiple comparisons. *p < 0.05, **p < 0.01, ***p < 0.001.

As shown in Figure 4, after three rounds of rating, the average GQS scores for dietary weight loss videos on BiliBili, TikTok, and Kwai were 2.04, 1.81, and 1.70, respectively, while the average mDISCERN scores were 2.01, 1.81, and 1.73. The median scores for both GQS and mDISCERN across the three platforms were 2. Although BiliBili scored higher than those of Kwai and TikTok (GQS: *p* < 0.01, *p* = 0.02; mDISCERN: *p* = 0.02, *p* = 0.08), overall scores remained below 3 on the three platforms. According to the definition of GQS in Supplementary Table S2, a score of 5 indicates excellent quality, 4 indicates good quality, 3 indicates moderate quality, and 1–2 indicate poor quality. Therefore, the observed mean and median GQS values suggest that low-quality videos were predominant across all platforms. This result may be attributed to insufficient oversight of the scientific rationality and authenticity of their video content on these platforms.

To validate the consistency of our assessment, we analyzed the scoring agreement among the three primary professional raters during the first round of evaluation. Table 2 presents the mean and median scores assigned by each rater, alongside the corresponding agreement metrics. Notably, the analysis revealed exceptional consistency in the judgments of the professional raters, with all Fleiss Kappa coefficients exceeding 0.90 for both GQS and mDISCERN scores. This near-perfect agreement confirms the robustness and reliability of the quality assessment data used in this study.

**Table 2:** Comparisons of first-round scores and inter-rater agreement among three professional raters, with Fleiss’ Kappa analysis.

Rater	GQS			mDISCERN		
	BiliBili	TikTok	Kwai	BiliBili	TikTok	Kwai
Rater#1	2.08 (2)	1.90 (2)	1.87 (2)	1.95 (2)	1.81 (2)	1.86 (2)
Rater#2	2.26 (2)	1.84 (2)	1.63 (2)	2.18 (2)	1.84 (2)	1.73 (2)
Rater#3	2.32 (2)	2.02 (2)	1.63 (2)	2.24 (2)	2.48 (3)	2.17 (2)
Fleiss Kappa	0.90	0.95	0.98	0.90	0.93	0.94

Data are presented as Mean (Median).

#### 3.2.2 Comparison between professional and non-professional raters

Table 3 compares the average GQS and mDISCERN scores assigned by professional and non-professional raters across the three platforms. The results indicated poor agreement between experts and the general audience regarding the quality and reliability of dietary weight loss videos, with the highest Cohen’s Kappa coefficient being less than 0.2.

**Table 3:** Comparison of scores between professional and non-professional raters, with Cohen’s Kappa analysis.

Rater type	GQS			mDISCERN		
	BiliBili	TikTok	Kwai	BiliBili	TikTok	Kwai
Professional	2.04 (2)	1.81 (2)	1.70 (2)	2.01 (2)	1.81 (2)	1.73 (2)
Non-professional	2.10 (2)	2.29 (2)	2.77 (2)	1.67 (2)	1.91 (2)	2.41 (3)
Cohen’s Kappa	0.19	0.05	0.04	0.13	0.12	0.03

Data are presented as Mean (Median).

To further characterize the sources of this divergence, we examined agreement across individual dimensions of the mDISCERN scale. For BiliBili videos, the highest agreement was observed in the Clarity dimension (Cohen’s *κ* = 0.43), whereas the greatest disagreement occurred in Fairness (Cohen’s κ = −0.02). On TikTok, the highest agreement was also found in the Clarity dimension (Cohen’s κ = 0.14), while the largest divergence was observed in the Reliability dimension (Cohen’s κ = −0.09). For Kwai, agreement remained consistently low across all dimensions, with the highest Cohen’s κ reaching only 0.05 (Reliability) and the lowest being −0.02 (References and Rigor). Detailed results are presented in Supplementary Table S3.

This suggests that raters tend to reach higher agreement when assessing whether a video clearly conveys its intended message (Clarity), whereas evaluating whether the content is balanced, accurate, and evidence-based (Reliability or Fairness) requires more professional expertise, leading to lower agreement. We also reviewed a subset of highly discordant videos on BiliBili for qualitative analysis, whose results supported this observation. Specifically, these videos frequently featured popular dietary regimens, such as the ‘ketogenic diet,’ emphasizing rapid weight loss effects while omitting potential adverse effects or medical contraindications. Non-professional raters, lacking the medical background to detect these omissions, tended to rate such content highly based on its persuasive narrative and promised efficacy.

#### 3.2.3 Comparison across different categories

As shown in Table 4, the relationship between uploader type and video quality differed markedly across platforms. On BiliBili, videos published by professional doctors were rated significantly lower than those by non-doctors in both GQS (1.56 vs. 2.13, *p* < 0.05) and mDISCERN (1.50 vs. 2.10, *p* < 0.05). In contrast, on TikTok, doctor-uploaded videos consistently received higher quality and reliability scores than non-doctor videos, particularly in mDISCERN (2.60 vs. 1.77; *p* < 0.05). On Kwai, the pattern was less consistent: doctor videos scored slightly lower than non-doctor videos on GQS (1.65 vs. 1.71, *p* = 0.68) but demonstrated higher reliability as reflected by mDISCERN scores (2.05 vs. 1.65; *p* < 0.05). These contrasting patterns indicate substantial platform-specific differences in the quality performance of dietary weight loss videos uploaded by doctors.

**Table 4:** Comparison of scores for videos from doctor and non-doctor creators across the three platforms.

Uploader type	GQS			mDISCERN		
	BiliBili	TikTok	Kwai	BiliBili	TikTok	Kwai
Doctor	1.56	2.00	1.65	1.50	2.60	2.05
Non-doctor	2.13	1.80	1.71	2.10	1.77	1.65

**Figure 5:**
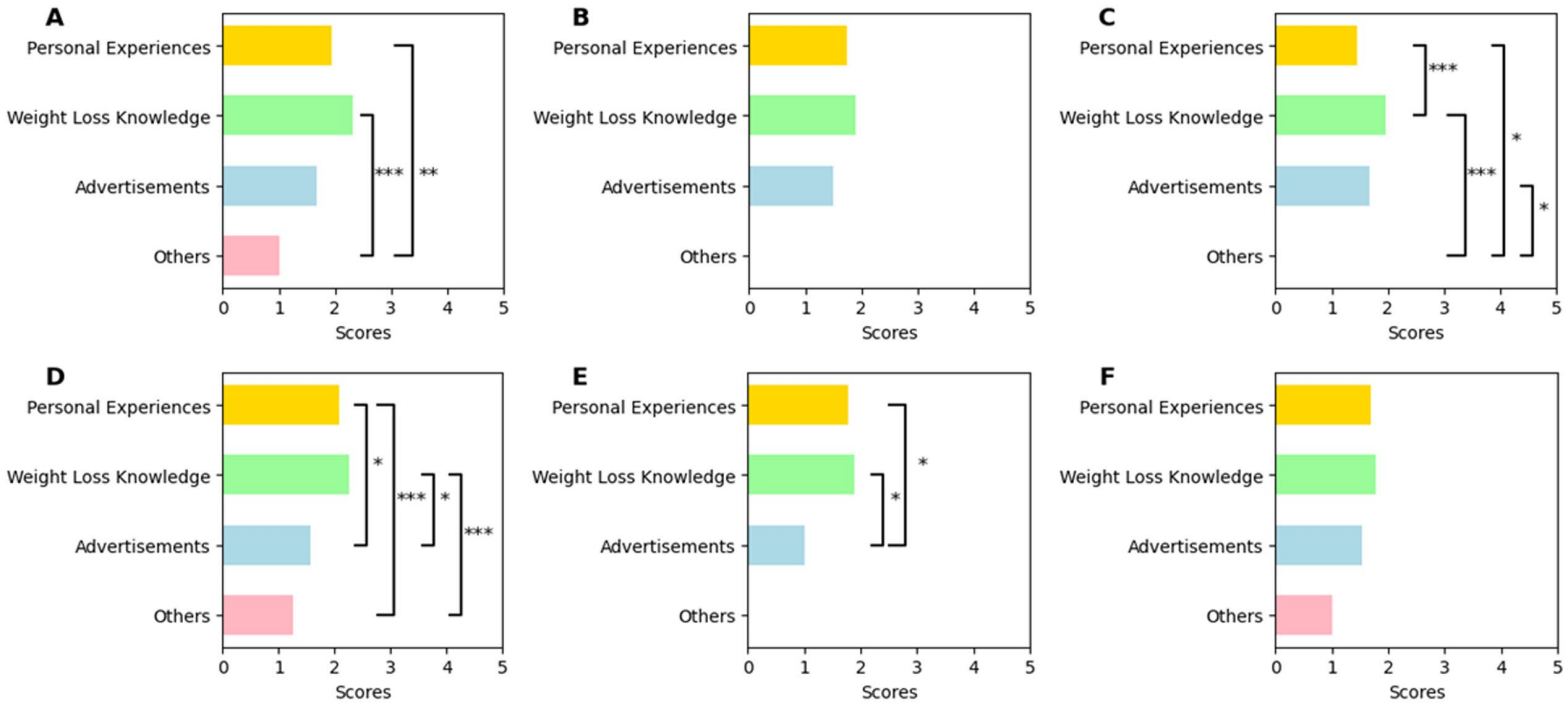
Comparison of video scores in different content categories. The first row presents the mDISCERN scores of **(A)** BiliBili, **(B)** TikTok, and **(C)** Kwai, respectively. The second row presents the GQS scores of **(D)** BiliBili, **(E)** TikTok, and **(F)** Kwai, respectively. Pairwise comparisons between platforms were performed using Mann–Whitney *U* tests and *p*-values were unadjusted for multiple comparisons. *p < 0.05; **p < 0.01; ***p < 0.001.

Table 5 shows that videos focused on weight loss knowledge received the highest GQS and mDISCERN scores across all platforms. Personal experience videos ranked second, except for the mDISCERN scores on Kwai, where advertisement videos held the second-highest position. As TikTok did not have videos int. the other category, the corresponding items in the table are marked by “/.” Figure 5 visualizes the comparison among four content categories with significance. Although weight loss knowledge videos were highest in all groups, it had no significant difference from the second place.

**Table 5:** Comparison of score for videos with different contents.

Content type	GQS			mDISCERN		
	BiliBili	TikTok	Kwai	BiliBili	TikTok	Kwai
Personal experiences	2.07	1.78	1.70	1.93	1.74	1.44
Weight loss knowledge	**2.26**	**1.88**	**1.77**	**2.31**	**1.90**	**1.95**
Advertisements	1.56	1.00	1.53	1.67	1.50	1.67
Others	1.25	/	1.00	1.00	/	0.00

The highest score of each column is in bold. “/” means that no samples in the corresponding category.

### 3.3 Correlation analysis

Consistent with our *priori* hypotheses, video duration was positively correlated with quality scores, whereas engagement metrics generally showed negative associations. Positive correlations were observed between GQS scores and video duration (*r* = 0.41, *p* < 0.01), as well as between mDISCERN scores and video duration (*r* = 0.32, *p* < 0.01). Strong positive correlations were also found between the number of likes and saves (*r* = 0.90, *p* < 0.01), likes and comments (*r* = 0.92, *p* < 0.01), and saves and comments (*r* = 0.86, *p* < 0.01). In contrast, negative correlations were observed between GQS scores and the number of likes, saves, and comments (*r* = −0.18, *p* < 0.01; *r* = −0.10, *p* = 0.09; and *r* = −0.17, *p* < 0.01, respectively). Similarly, mDISCERN scores were negatively correlated with likes, saves, and comments (*r* = −0.12, *p* < 0.05; *r* = −0.02, *p* = 0.77; and *r* = −0.13, *p* < 0.05). Video duration was also negatively correlated with the number of likes, saves, and comments (*r* = −0.36, *r* = −0.22, and *r* = −0.32, respectively; all *p* < 0.01).

In addition, we also conducted multivariate regression analyses including video duration, engagement metrics, uploader type, and content category as predictors. Overall, the associations between video characteristics and quality scores varied across platforms. Supplementary Table S4 presents regression results for BiliBili. Both GQS and mDISCERN scores were negatively associated with uploader type (GQS: *β* = −0.72, *p* < 0.001; mDISCERN: *β* = −0.76, *p* < 0.01) but positively associated with content category (GQS: *β* = 0.55, *p* < 0.001; mDISCERN: *β* = 0.67, *p* < 0.001), with longer videos also showing higher mDISCERN scores (*β* = 0.21, *p* < 0.05). The adjusted R^2^ for GQS and mDISCERN were 0.21 and 0.24, respectively. In contrast, as shown in Supplementary Table S5, video duration on TikTok was positively associated with GQS scores (*β* = 0.23, *p* < 0.001), while mDISCERN scores were positively associated with both uploader type (*β* = 0.75, *p* < 0.01) and video duration (*β* = 0.11, *p* < 0.05). The adjusted R^2^ for GQS and mDISCERN were 0.15 and 0.11, respectively. For Kwai, Supplementary Table S6 indicates that GQS scores increased with video duration (*β* = 0.20, *p* < 0.01) and the number of saves (*β* = 0.19, *p* < 0.05), but decreased with the number of likes (*β* = −0.30, *p* < 0.01). Meanwhile, mDISCERN scores on Kwai were positively associated with uploader type (*β* = 0.34, *p* < 0.05), content category (*β* = 0.43, *p* < 0.01), and video duration (*β* = 0.14, *p* < 0.05). The adjusted R^2^ for GQS and mDISCERN were 0.14 and 0.24, respectively. Although strong correlations among likes, saves, and comments, only likes (VIF = 7.18) and comments (VIF = 6.86) on TikTok showed moderate multicollinearity.

## 4 Discussion

### 4.1 Principal findings

In this cross-sectional study, we collected and reviewed videos on the topic of dietary weight loss from the three most popular video platforms in China, namely BiliBili, TikTok, and Kwai. Despite BiliBili achieving statistically higher scores than TikTok and Kwai, the mean scores across all platforms remained below 3.0, indicating a predominance of low-quality content. Our multivariate analysis revealed three critical trends. First, a trade-off exists between quality and engagement: video duration was a positive predictor of quality but was negatively correlated with popularity metrics, suggesting that algorithms algorithm-driven popularity often favors shorter, less reliable content. Second, the impact of professional sources was platform-dependent; surprisingly, doctor uploader status was positively associated with quality on TikTok but negatively associated on BiliBili. Third, there was poor agreement between professional and lay raters (Cohen’s *κ* < 0.2). While lay audiences could assess content clarity, they struggled to distinguish narrative appeal from scientific reliability, highlighting a significant gap in digital health literacy.

### 4.2 Video source, video content and video quality

First, we observed a substantial divergence between professional and non-professional raters across the three platforms. For professional raters, the GQS and mDISCERN scores were ranked from highest to lowest as BiliBili, TikTok, and Kwai. In contrast, non-professional raters ranked the platforms from highest to lowest as Kwai, TikTok, and BiliBili. Our dimension-specific analysis (Supplementary Table S3) implies that the discrepancy likely stems from Fairness and Reliability. This low agreement underscores a critical health communication issue: a fundamental disconnect exists between “expert-driven assessment” and “user-perceived value”. Therefore, video creators should be aware of the urgent need for health communication strategies that go beyond content accuracy to address how users actually consume and value information.

Second, we found that videos from BiliBili published by professional doctors scored lower on both the GQS and mDISCERN scales compared to those by non-doctors, which is contrary to previous research findings (19, 21). This divergence may be attributed to topic specificity and information delivery styles. Unlike disease-related topics requiring a strong professional background, dietary weight loss is a lifestyle-integrated topic where non-professional creators often employ narrative-based communication. By blending personal weight-loss journeys with practical tips, these creators enhance the “utility” and “flow” of information, making the videos more accessible to audience. In contrast, videos by professional doctors are more didactic, lacking the same personalized appeal. Moreover, BiliBili videos were substantially longer than those on TikTok and Kwai, which likely increases the demand for the narrative organization ability. If doctors are less experienced in digital content delivery, these limitations could be more salient in long-duration videos.

Third, regarding content categories, videos focusing on “Weight Loss Knowledge” outperformed other three categories across all platforms. Personal experiences with dietary weight loss might spread misinformation, such as losing weight by eating only a cucumber at night or not eating at all and how much weight was lost as a result. In contrast, videos sharing weight loss knowledge were based on scientific evidence, such as the “16 + 8 diet” or “ketogenic diet,” naturally scoring higher than other types of content.

### 4.3 Comparison with prior research frameworks

In the evolving landscape of social media health analysis, GQS and mDISCERN have emerged as mainstream instruments. Although originally developed for text-based websites, GQS was selected for its holistic focus on “flow,” “ease of understanding,” and “overall usefulness”—qualities well aligned with the rapid, intuitive consumption of short videos. Specifically, GQS has been widely adopted in recent assessments of short videos regarding immune-mediated inflammatory disease (19, 26, 30), gallstone disease (22), cancer (21, 27, 31), and bariatric surgery (23, 32). DISCERN is also a frequently-used instrument in medical video assessment task (20, 22, 24, 26, 33). However, its 16-item structure is often considered too lengthy for rapid videos analysis, thus we utilized the adapted mDISCERN for greater efficiency. However, this choice entails a methodological limitation: other validated instruments such as PEMAT (34), JAMA (22, 23, 32), and HONcode (33) were not employed.

Beyond the choice of instruments, our study expands upon existing procedural paradigms in two key aspects. First, unlike the majority of prior research which typically limits investigation to a single platform (e.g., TikTok only) (21, 23, 26, 27, 35) or dual platforms (21, 36), our study conducts a comprehensive comparative analysis across China’s three dominant video platforms (BiliBili, TikTok, and Kwai). This multi-platform approach offers a broader ecological view of the digital information landscape. Second, distinct from the standard practice of relying exclusively on medical professionals for scoring, our experimental design explicitly incorporated non-professional raters. This inclusion addresses a methodological limitation in previous works by simulating the actual perspective of the lay public, thereby enhancing the ecological validity of the quality assessment.

When comparing our results with these prior frameworks, a distinct “domain-specific risk” becomes evident. Our analysis revealed that dietary weight loss videos generally failed to achieve satisfactory quality scores (median scores = 2; mean scores in the range of 1.7–2.1). These values are systematically lower than those reported in studies of more domain-specific topics on the same platforms. For instance, on TikTok, videos related to thyroid cancer achieved a mean GQS of 3.72 (35) and videos regarding liver cancer reached a median GQS of 3 (21). Notably, this quality gap persists even when compared to dietary guidance for specific clinical conditions. Our scores were lower than those reported for inflammatory bowel disease dietary management on Chinese platforms (mean GQS 2.61, median 3) (19) and post-bariatric surgery nutrition (mean GQS 2.35, median 2) (23). This discrepancy suggests that unlike oncology or surgery content, which retains a higher barrier to entry, the dietary weight loss domain is disproportionately susceptible to low-quality information and lacks the rigor seen in other medical specialties.

### 4.4 Public health implications

Over the past forty years, more than half of Chinese adult population has become overweight or even obese (37). Obesity can lead to a range of metabolic disorders such as hypertension, hyperglycemia, and hyperuricemia, which pose a serious threat to public health. A multitude of evidence suggests that a weight loss of 5–15% can significantly improve metabolic disorders and reduce the risk of cardiovascular diseases and other weight-related comorbidities (38, 39). As the primary source of health information shifts from medical professionals to the internet, the uneven quality of online content poses new challenges. Low-quality diet videos may spread misinformation and encourage unhealthy behaviors, such as crash dieting or following unbalanced regimens (40, 41).

From a health communication perspective, lifestyle-oriented topics are more conducive to narrative and experience-based communication styles that enhance relatability and audience engagement but may compromise informational rigor. In addition, from the standpoint of digital health literacy frameworks, audiences may possess adequate functional literacy to understand practical dietary advice while lacking the critical literacy required to assess scientific credibility. Collectively, these factors may make the dietary weight loss domain particularly susceptible to the spread of lower-quality information.

Crucially, our study revealed a negative correlation between video quality and engagement metrics. This phenomenon is likely driven by the inherent nature of short-video platforms: users typically consume content during fragmented leisure time, favoring shorter, fast-paced videos. Consequently, algorithmic recommendation mechanisms may be associated with greater visibility for videos with higher completion rates. However, given the cross-sectional nature of our study, these findings should be interpreted as associations only and do not establish a causal relationship between algorithmic recommendation mechanisms and video quality. This pattern may suggest the presence of a “popularity bias,” which may restrict the effective dissemination of evidence-based information to the intended audience. Addressing this potential imbalance may require coordinated efforts at multiple levels.

First, at the policy level, building on the Chinese government’s issuance of the first health promotion guide (42), we recommend that health authorities strengthen the implementation of existing policies by developing clear standards for short-video health content, establishing evaluation and certification mechanisms, and enhancing supervision of misleading information. Second, video platforms should take proactive steps by establishing dedicated medical sections, forming expert review teams to assess medical content, implementing visible credibility indicators (e.g., verified professional badges), and refining video recommendation algorithms to prioritize content quality over mere engagement. Additionally, medical professionals need to improve their digital communication skills and be responsible for the accuracy and reliability of the content they produce. Finally, beyond regulation, there is an urgent need for digital health literacy interventions. Public health education should focus on equipping individuals with critical viewing skills, enabling them to distinguish between scientific advice and misleading narratives in the complex digital environment.

### 4.5 Limitations and future work

Our study has several limitations. First, the selection of the top 100 videos based on platform algorithms likely introduces an inherent selection bias. While this sampling method effectively captures the information most accessible to the general public, it limits the generalizability of our results to the broader corpus of dietary weight-loss videos. High-quality educational videos that fail to achieve high algorithmic rankings may have been excluded from this analysis. Second, only a single keyword was used to retrieve videos, and the potential use of multiple keywords from a user-centered perspective was not explored. Third, while data were collected within a single day to control for algorithmic volatility, this cross-sectional snapshot may not fully account for temporal fluctuations in ranking and recommendation systems. Fourth, our study focused videos from Chinese platforms and did not assess or compare contents from non-Chinese videos, which limits the generalizability of our findings to international video platforms. Fifth, as a content analysis, this study focuses solely on assessing the quality and reliability of the information provided. We did not investigate the actual impact of these videos on user outcomes, such as behavioral changes or weight loss efficacy. Finally, we acknowledge the statistical issue of multiplicity arising from the numerous comparisons performed. As an exploratory analysis, we did not apply adjustments (e.g., Bonferroni correction) to avoid masking potential signals, inherently inflating the risk of Type 1 errors. Therefore, marginal *p*-values should be interpreted with caution, and these findings warrant validation in future studies with larger sample sizes.

In future work, we aim to adopt a more user-centered approach to simulate real-world information retrieval behaviors. This approach will incorporate multiple relevant keywords (e.g., colloquial terms and synonyms) and adjust sampling strategies to focus on the top-ranked results. In addition, samples will be collected at different time points to account for temporal variations in algorithmic recommendations. Together, these strategies aim to capture the content users are most likely to encounter in real time. Such studies will complement our current landscape analysis and better reflect the diversity of dietary weight-loss information accessible to the public.

## 5 Conclusion

We collected 100 videos on the theme of dietary weight loss from BiliBili, TikTok and Kwai, and evaluated their reliability and quality using the GQS and mDISCERN tools. After three rounds of scoring, we found that while the GQS and mDISCERN scores for BiliBili were higher than those of TikTok and Kwai, the final scores of the three platforms did not exceed 3 points, which indicated that the video quality of the three video platforms on the theme of dietary weight loss was low. Medical practitioners are encouraged to actively create high-quality medical science popularization videos, providing reliable sources of information for public health education. At the same time, video social media platforms should establish relevant policies to supervise and review the publication of medical science popularization videos, in order to prevent users from receiving incorrect medical knowledge. These combined efforts are important for improving digital health communication and promoting effective dissemination of dietary weight loss information in the digital environment.

## Data Availability

The raw data supporting the conclusions of this article will be made available by the authors, without undue reservation.
