# Supplementary material for: Reliability and quality assessment of internet videos as guidance for dietary weight loss intervention: a cross-sectional study in China
Source: Front Public Health. 2026 Mar 19;14:1749849. doi: 10.3389/fpubh.2026.1749849 (PMC13044112; doi:10.3389/fpubh.2026.1749849)
Supplement: Supplementary file 1 [file Table_1.DOCX]

**Table S1.** Chinese and English Versions of the mDISCERN Rating Scale. The Chinese version is adopted in our study while the English translations are provided for non-Chinese-speaking readers.

| **mDISCERN** | **English Description** | **Chinese Description** |
| --- | --- | --- |
| Clarity | Is the video clear, concise, and understandable? | 视频是否清晰、简洁、易懂？ |
| Reliability | Are valid sources cited? | 视频是否引用了有效的来源？ |
| Fairness | Is the content presented balanced and unbiased? | 视频所呈现的内容是否平衡公正且无偏见？ |
| References | Are additional sources of content listed for patient reference? | 视频是否列出了其他信息来源以供患者参考？ |
| Rigor | Are areas of uncertainty mentioned? | 视频是否提及了不确定性领域？ |

**Table S2.** Chinese and English Versions of the GQS Rating Scale. The Chinese version is adopted in our study while the English translations are provided for non-Chinese-speaking readers.

| **Level** | **English Description** | **Chinese Description** |
| --- | --- | --- |
| 1 | Poor quality, poor flow of the site, most information missing, not at all useful for patients | 视频质量差，结构混乱，大部分信息缺失，对患者完全无用。 |
| 2 | Generally poor quality and poor flow, some information listed but many important topics missing, of very limited use to patients | 视频总体质量较差且结构混乱，虽然列出了部分信息但是缺少重要的论题，对患者的实际帮助极为有限。 |
| 3 | Moderate quality, suboptimal flow, some important information is adequately discussed but others poorly discussed, somewhat useful for patients | 视频质量中等，结构不够理想，一些重要信息讨论得比较充分，但其他信息讨论不够，对患者有一定帮助。 |
| 4 | Good quality and generally good flow, most of the relevant information is listed, but some topics not covered, useful for patients | 视频质量较好，整体结构合理，大部分相关信息都有涵盖，但部分内容未涉及，对患者有用。 |
| 5 | Excellent quality and excellent flow, very useful for patients | 视频质量极高，结构非常合理，对患者非常有帮助。 |

**Table S3.** Cohen’s Kappa Analysis of mDISCERN Scores Across Different Dimensions for Professional and Non-Professional Raters.

| **Dimension** | BiliBili | TikTok | Kwai |
| --- | --- | --- | --- |
| Clarity | 0.43 | 0.14 | 0.03 |
| Reliability | 0.29 | -0.09 | 0.05 |
| Fairness | -0.02 | 0.10 | 0.04 |
| References | 0.32 | 0.00 | -0.02 |
| Rigor | 0.11 | 0.00 | -0.02 |

**Table S4.** Summary of Multivariate Linear Regression Models for BiliBili Videos. Bolded values indicate P < 0.05.

|  | VIF | Score | β | SE | 95% CI | P-value |
| --- | --- | --- | --- | --- | --- | --- |
| Uploader^a^ | 1.05 | GQS | **-0.72** | 0.19 | (-1.10, -0.35) | <0.001 |
|  |  | mDISCERN | **-0.76** | 0.23 | (-1.21,-0.31) | <0.01 |
| Content^b^ | 1.09 | GQS | **0.55** | 0.15 | (0.25,0.84) | <0.001 |
|  |  | mDISCERN | **0.67** | 0.18 | (0.32,1.03) | <0.001 |
| Duration | 1.03 | GQS | 0.11 | 0.07 | (-0.04,0.25) | 0.15 |
|  |  | mDISCERN | **0.21** | 0.09 | (0.04,0.38) | <0.05 |
| Like | 2.09 | GQS | -0.10 | 0.10 | (-0.30,0.11) | 0.36 |
|  |  | mDISCERN | -0.22 | 0.12 | (-0.47,0.02) | 0.08 |
| Save | 1.26 | GQS | -0.04 | 0.08 | (-0.20,0.12) | 0.60 |
|  |  | mDISCERN | 0.00 | 0.10 | (-0.20,0.19) | 0.96 |
| Comment | 1.87 | GQS | 0.00 | 0.10 | (-0.19,0.20) | 0.98 |
|  |  | mDISCERN | 0.07 | 0.12 | (-0.16,0.31) | 0.53 |

^a^ Reference group: Non-doctor.

^b^ Reference group: Non-knowledge content (Personal experiences, Advertisements, Others)

**Table S5.** Summary of Multivariate Linear Regression Models for TikTok Videos. Bolded values indicate P < 0.05.

|  | VIF | Score | β | SE | 95% CI | P-value |
| --- | --- | --- | --- | --- | --- | --- |
| Uploader^a^ | 1.12 | GQS | 0.10 | 0.24 | (-0.39,0.58) | 0.70 |
|  |  | mDISCERN | **0.75** | 0.26 | (0.23,1.26) | <0.01 |
| Content^b^ | 1.10 | GQS | 0.19 | 0.11 | (-0.02,0.40) | 0.08 |
|  |  | mDISCERN | 0.11 | 0.11 | (-0.11,0.34) | 0.31 |
| Duration | 1.04 | GQS | **0.23** | 0.05 | (0.13,0.34) | <0.001 |
|  |  | mDISCERN | **0.11** | 0.05 | (0.01,0.22) | <0.05 |
| Like | 7.18 | GQS | 0.01 | 0.13 | (-0.25,0.28) | 0.92 |
|  |  | mDISCERN | 0.01 | 0.14 | (-0.27,0.29) | 0.93 |
| Save | 1.47 | GQS | 0.01 | 0.06 | (-0.12,0.13) | 0.94 |
|  |  | mDISCERN | 0.06 | 0.06 | (-0.07,0.19) | 0.35 |
| Comment | 6.86 | GQS | -0.06 | 0.13 | (-0.32,0.20) | 0.63 |
|  |  | mDISCERN | -0.01 | 0.14 | (-0.29,0.26) | 0.93 |

^a^ Reference group: Non-doctor.

^b^ Reference group: Non-knowledge content (Personal experiences, Advertisements, Others)

**Table S6.** Summary of Multivariate Linear Regression Models for Kwai Videos. Bolded values indicate P < 0.05.

|  | VIF | Score | β | SE | 95% CI | P-value |
| --- | --- | --- | --- | --- | --- | --- |
| Uploader^a^ | 1.13 | GQS | -0.12 | 0.15 | (-0.42,0.17) | 0.41 |
|  |  | mDISCERN | **0.34** | 0.16 | (0.02,0.66) | <0.05 |
| Content^b^ | 1.14 | GQS | 0.13 | 0.12 | (-0.12,0.37) | 0.30 |
|  |  | mDISCERN | **0.43** | 0.13 | (0.17,0.69) | <0.01 |
| Duration | 1.04 | GQS | **0.20** | 0.06 | (0.09,0.32) | <0.01 |
|  |  | mDISCERN | **0.14** | 0.06 | (0.01,0.26) | <0.05 |
| Like | 3.86 | GQS | **-0.30** | 0.11 | (-0.52,-0.08) | <0.01 |
|  |  | mDISCERN | **-0.20** | 0.12 | (-0.44,0.03) | 0.09 |
| Save | 2.43 | GQS | **0.19** | 0.09 | (0.01,0.37) | <0.05 |
|  |  | mDISCERN | 0.12 | 0.09 | (-0.07,0.31) | 0.20 |
| Comment | 1.73 | GQS | 0.08 | 0.07 | (-0.07,0.23) | 0.29 |
|  |  | mDISCERN | -0.06 | 0.08 | (-0.22,0.10) | 0.43 |

^a^ Reference group: Non-doctor.

^b^ Reference group: Non-knowledge content (Personal experiences, Advertisements, Others)
